# Practical Observations on the Nature and Treatment of Tuberculosis of the Hip-Joint

**Published:** 1858-07

**Authors:** S. D. Gross

**Affiliations:** Professor of Surgery in the Jefferson Medical College of Philadelphia


### ART. I.—*Practical Observations on the Nature and Treatment of Tuberculosis of the Hip-joint, commonly called Coxalgia.* By S. D. GROSS, M.D., Professor of Surgery in the Jefferson Medical College of Philadelphia.

FROM the earliest ages of medicine, hip-joint disease has been an object of the deepest interest with the practitioner. Its literature, scattered through an immense number of monographs, cyclopædias, and periodicals, is exceedingly rich, and fully attests the truth of this remark; and yet, notwithstanding this, it is lamentable to think how imperfectly it is still understood by the mass of the profession; how little is known about its nature and etiology; and how much remains to be done to place it upon a proper scientific basis.

The term coxalgia, by which this disease is usually described, should be discarded from surgical nomenclature, as wholly inexpressive of its true character. Literally signifying pain, or neuralgia suffering, it is neither denotive of its pathology, nor, indeed, even always of its seat; for, as will hereafter be seen, the pain is nearly as often situated in other parts as in the hip-joint itself. The words hip-disease, hip-joint disease, and coxarthrocace are not less indefinite, and should therefore be equally proscribed. The only appropriate name is strumous, tubercular, or scrofulous, the import of which is well understood, and which could not possibly more clearly and satisfactorily express the true nature of the complaint. I make these remarks because the nomenclature of a disease is frequently of great importance in regard to the proper appreciation of its pathology.

Strumous disease of the hip-joint occurs at different periods of life; but the age at which it usually shows itself is early childhood. As far as my experience goes, and I do not hesitate to say that it is very large, I am satisfied that it is by far most common from the third to the eleventh year. Cases of it are occasionally witnessed as early as the eighteenth month, and a good many just before the time of puberty. After the twentieth year the malady is uncommon; and, if an instance is sometimes met with in middle life, it is to be regarded as an exception to what must be considered as the great law which presides over the development of this form of strumous disease. Tuberculosis of the lungs is most common between the ages of twenty and forty; of the lymphatic ganglions, the spleen, eye, and arachnoid membrane, between the ages of three and ten. Every organ in which the malady occurs has, it may be presumed, its peculiar laws of evolution, growth, maturation, and disintegration of the deposit from which the affection derives its distinctive features. That this is true of the hip-joint, as it is of other parts of the body, all experience attests. I recollect only one case of tuberculosis of this articulation after the thirtieth year.

The malady is met with in both sexes, but in what proportion has not been determined, as no statistics, illustrative of the subject, have been published. My observation has furnished me with a much larger number of cases in the male than the female; but this may have been a mere coincidence, and the question, after all, is one of no practical value, whatever may be the fact with reference to it.

Tuberculosis of the hip-joint is occasionally observed in several members of the same family. The instances of this, however, are rare, and I have not seen more than three in my own practice and in that of my medical friends. If it is ever congenital I have not witnessed an example of it; nor have I ever known it to be hereditary, although many of my cases have occurred in the offspring of strumous parents, or in families in which tuberculosis had manifested itself in some form or other. In this respect it bears, as will appear by and by, the same relation to the structures of the hip-joint that phthisis bears to those of the lungs.

### I.—ETIOLOGY.

The exciting causes of this affection have been a prolific source of speculation, of dispute, and even of angry discussion; and, still, the most that can, with truth, be said of them is, that they are, in general, exceedingly obscure. In the great majority of cases, it arises spontaneously, defying all conjecture in respect to its origin. The surgeon, it is true, is often told that the little patient received a fall, blow, or kick upon the affected parts, or that the joint was sprained, bruised, or twisted, several months, perhaps, before the appearance of the characteristic symptoms; but my experience is that such information is usually little reliable, or that, if the occurrence did take place as stated, it excited but little if any influence in developing the malady. Unless very severe, such accidents would no more occasion tuberculosis of the hip-joint than a similar injury of the chest would produce tuberculosis of the lungs, or of the head tuberculosis of the arachnoid membrane. The possibility of such an occurrence cannot, of course, be denied, but it may be safely affirmed that it is infrequent, and therefore exceptional.

Exposure to cold, intense or protracted, is usually enumerated as another exciting cause of the malady, and generally with a better show of reason. That it may, in such an event, conduce to this result, especially in a child of feeble constitution, ill-fed, and with an impoverished state of the blood, hardly admits of doubt. It is well known that cold is one of the most common and powerful causes of pulmonary consumption and of strumous disease of the neck. Living in damp, under-ground, and ill-ventilated apartments operates in the same manner. Simple suppression of the cutaneous perspiration, suddenly induced, as when a person is exposed to a heavy current of air, might, it may be presumed, easily occasion the affection in one predisposed to its occurrence.

The use of unwholesome food, derangement of the digestive organs, imperfect assimilation, or inadequate nutrition, however induced, are also among the causes of this disease, as they are among the causes of tuberculosis in general. Protracted courses of mercury, establishing a severe drain upon the system, followed by the abstraction of the plastic elements of the blood, may lead to similar results. The same is true, though the circumstance does not always readily admit of proof, of the exhaustion consequent upon copious and protracted hemorrhages, infantile cholera, chronic diarrhœa, scarlatina, measles, smallpox, and of typhoid, intermittent, and other fevers; in short, of everything that has a tendency to enfeeble the system and degrade the blood.

It has been asserted by writers on coxalgia that rheumatism is a common cause of this disease, but I have never seen an instance corroborative of the truth of the statement. The fact is, it is not at all probable that that affection ever exerts such an influence; for, in the first place, it is well known that tuberculosis is exceedingly rare in rheumatic subjects, and, in the second, that, when disease of the hip-joint shows itself in persons of this description, it is very different from the strumous disorder under consideration.

Persons of fair complexion, light hair and eyes, a delicate skin, and a languid circulation, with a tendency to eruptions of the scalp and enlargement of the lymphatic ganglions, are most prone to tuberculosis of the hip-joint. In many cases the strumous diathesis exists in a most marked degree, the tumid lip and belly, the long eyelashes, the cold extremities, the flattened shape of the fingers, and the disordered condition of the digestive apparatus, affording unmistakable evidence of its presence. The intellectual faculties vary in this as in other forms of strumous disease. Occasionally the little patient is dull and obtuse, with little disposition to exertion; but for the most part he is remarkably precocious and active, both in mind and body, inclined to exercise and amusement even long after the malady has been fully established. No one who has frequently met with this affection can have failed to notice the different temperaments of those who are most liable to suffer from it. These are the sanguine and the lymphatic, or a combination of these. In the former the characteristics are, a rosy state of the countenance, a well-developed muscular system, with a tendency frequently to a certain degree of embonpoint, an active circulation of the skin, warmth of the extremities, and an active state of the intellect. Opposed to this, in every particular, is the lymphatic temperament. The face is pale, often swollen and pasty, the muscles are soft and flabby, the feet are cold, the cutaneous circulation is feeble, the mind is sluggish, and the pupil is dilated. In both, particularly in the latter, the belly and upper lip are often remarkably tumid, and most expressive of the tubercular diathesis. These two varieties of temperament, with their various modifications, deserve careful consideration, as they form the basis of important therapeutic indications.

It is rare that both hip-joints are involved in this affection, either simultaneously or consecutively. I do not now recollect an instance of the kind within the sphere of my observation. During its progress it becomes occasionally complicated with tuberculosis of the spine, as Pott's disease and psoas abscess, ophthalmitis, mesenteric disease, strumous enlargement of the lymphatic ganglions of the neck, and pulmonary phthisis. It is not impossible that some of these affections may occasionally precede the malady of the joint; but, however this may be, they rarely fail, when present, to aggravate it, and to hasten the fatal issue. In proof of the truth of this fact several cases will hereafter be cited from my own practice.

### II.—SYMPTOMATOLOGY.

Tuberculosis of the hip-joint may be described as consisting of three stages, each characterized by distinctive symptoms and pathological changes, as well as requiring peculiar treatment. As this division is not imaginary but real, it is deserving of the greatest attention.

The symptoms by which the disease is usually announced are of so obscure and stealthy a character as to render it very liable to be mistaken for other affections of the joint. The first circumstance which commonly attracts attention, especially if the patient be a child, is a feeling of fatigue after exercise, with slight pain in the knee, and a disposition to drag the limb, thus giving the gait a stiff, awkward appearance. In this manner the case may progress for several weeks, or, indeed, even for several months, with, perhaps, hardly any perceptible aggravation. The child still goes about, taking his accustomed exercise, and manifesting the same interest as formerly in his out-door amusements. Gradually, however, the pain increases; there is now a distinct limping, and the sleep at night is apt to be disturbed by spasmodic twitches of the extremity. The pain is usually referred to the knee, particularly to its inner side, and is either sharp and lancinating, or dull, heavy, and aching. It is sometimes felt in the very depth of the joint, but more frequently it is superficial, as it were just beneath the integuments. Exercise, or motion of any kind, always increases it, and it is generally worse at night than in the day; damp states of the atmosphere, suppression of the cutaneous perspiration, and disorder of the digestive organs also frequently aggravate it. The knee, on inspection, is found to be free from swelling and discoloration, and commonly quite tolerant of rough manipulation, as motion, pressure, and percussion. Occasionally the pain is of a neuralgic nature, and distinctly periodical in its occurrence, very similar, in this respect, to the paroxysms of an intermittent fever; the attack, perhaps, coming on early in the evening, and, after having continued for a few hours, returning about the same time the next day. This form of pain is most frequent, as far as my observation enables me to judge, in persons living in a malarial atmosphere.

It is not often, however, that the pain, whatever be its character, is confined entirely, at this stage of the disease, to the knee; or, if it be so at first, that it remains there exclusively for any length of time. In general it extends also to the thigh and leg, sometimes along the front, now along the sides, especially the inner, and now along the posterior surface, in the direction, apparently, of some nervous trunk, as the crural, obturator, saphenous, or sacro-sciatic. I have known cases where the pain was felt most keenly at the tendo-achillis, just above the ankle-joint, and in one instance, under my charge not long ago, it was distressingly severe over the instep. Sometimes, again, the pain seems to shift from one of these points to another, being most severe, perhaps, at one time in the knee, and at another in the thigh, leg, or foot. It is proper also to add that the pain is generally not persistent, and that the patient has frequently long intervals of ease or of comparative comfort.

Various explanations have been offered respecting the occurrence of pain at the knee, but I cannot say that any of them, so far as it is in my power to appreciate them, afford any satisfactory solution. It has been supposed, for example, that it is owing to an inflamed condition of some of the principal nerves of the limb, especially the obturator, which, as is well known, occasionally sends a small filament to the hip-joint; but what connection has this nerve with the knee? None whatever; and the same is true of the other nerves of the lower extremity. Nor, in my judgment, is the opinion that it is owing to disease of the long head of the femoral muscle any more plausible. This muscle lies over, and is attached to, the capsular ligament of the hip-joint; but even supposing, what is not very probable at this early stage of the malady, that it participates in the morbid action, how could it give rise to the pain in the knee, the leg, and foot? Again, it has been imagined that the suffering in question is caused by inflammation of the cancellated structure of the head and neck of the thigh-bone; but if this be so, we have no positive proof of the fact. Our knowledge, then, in regard to this matter, is wholly conjectural, nothing more; a circumstance so much the less to be regretted, because it is really not of the least practical importance how the pain is produced, provided it is recollected that it usually manifests itself first at the knee. So far as this is concerned it is a symptom of great diagnostic value.

After some time, varying from a few weeks to as many months, the pain shifts to the hip and its neighborhood; or, if it do not entirely forsake the knee, it is generally less constant and severe there than it was in the first instance, or soon after the commencement of the morbid action. Commonly it is most intense and persistent directly over the articulation, deep-seated, and of a dull, gnawing character. At times it is perceived most keenly in the sacro-sciatic notch, between the great trochanter and the spine of the ilium, or in the upper and outer part of the groin. Occasionally, again, it exists simultaneously at all these points, although not in an equal degree; or, as it leaves one, it fastens itself upon another. In rare cases the pain appears in the hip before it shows itself in the knee, thigh, or leg. Pressure upon the gluteal region, motion of the affected joint, and percussion of the knee, the leg being flexed at a right angle, or of the sole of the foot, the limb being extended, always augments the pain, and leads to the detection of its seat.

As yet, there is no sensible impairment of the general health; the appetite is good, and the various tissues retain their normal development. The muscles of the affected hip and limb are, perhaps, a little thinner and softer than natural, but these changes are usually slight, and hence they often elude detection.

The symptoms now described may be regarded as, at least in some degree, characteristic of the first stage of hip-joint disease. Another train now succeeds, denotive of the steady advance of the morbid action, and equally significant of its presence.

The most prominent local phenomena, at this period, are, an increase of the pain in the hip and knee, flattening of the buttock, effacement of the gluteo-femoral crease, and apparent elongation of the limb, with spasmodic twitching and wasting of its muscles.

The pain, hitherto seated chiefly in the knee, now also affects the hip, or, if it existed there previously, as, indeed, is not unfrequently the case, it becomes sensibly aggravated. It is particularly violent at night, often for hours interrupting sleep, and attended with the most distressing spasmodic twitches of the muscles of the limb, which thus greatly augment the local and general suffering. The pain at one time is fixed, deep, aching, gnawing, or boring in its character; at another, erratic, sharp, or lancinating, darting about in different directions—now through the joint, then down the limb, and then through the groin, or back along the course of the sciatic nerve. Occasionally it is most severe in the lumbar region, in the lower part of the pelvis, in the situation of the acetabulum, or at the upper and inner part of the thigh. As before remarked, it is sometimes of a neuralgic character—coming, going, and recurring at particular periods. Derangement of the digestive apparatus, exposure to cold, and damp states of the atmosphere, have a tendency to aggravate and protract it. The pain in the knee, instead of disappearing, generally increases in violence, at the same time that it becomes more frequent and fixed.

The sleep is habitually disturbed by unpleasant dreams, and the patient often wakes up in great alarm, crying and screaming, being perhaps much bewildered, and unable to determine where he is or what is the matter with him. Occasionally he is partially delirious, and some minutes elapse before he regains his entire consciousness. He sleeps by snatches, and hence he usually feels fatigued and unrefreshed in the morning. Spasmodic twitching, jerking, or starting of the limb is a prominent symptom in this stage of the disease, and is rarely absent in any case Sometimes, indeed, it sets in at a very early period, and continues with more or less violence during the whole progress of the malady. It is particularly distressing in the muscles of the thigh, but often affects those also of the hip and leg.

Along with these symptoms are frequently, but by no means constantly, impairment of the appetite, and disorder of the secretions, with a certain amount of fever at night. The bowels are usually inclined to be constipated, the urine is scanty and high-colored, the skin is rather arid, especially in the forepart of the night, and the patient is disposed to drink more than common. As the case progresses the fever becomes more frequent and severe, and is often followed by pretty copious sweats. The patient loses flesh and strength, he is peevish and irritable, and his countenance has a care-worn appearance. Although such is ordinarily the state of the system, in the second stage, especially after the disease has made some progress, yet there are cases in which there is hardly any constitutional disturbance whatever, except what results from the loss of sleep.

The local phenomena at this stage of the malady are unmistakable. The buttock of the affected side is found to be remarkably flattened, so as to be in striking contrast with the sound one. It is much broader, as well as considerably larger than in the natural state; the gluteal muscles are soft and flabby, and the skin is preternaturally loose, apparently from the absorption of the subcutaneous adipose substance. The gluteo-femoral crease, which forms so prominent a feature of this part of the body in the natural state, is completely effaced, giving the thigh and hip an appearance of continuity, or as if they were fused together. The muscles of the thigh and leg are also wasted, and this circumstance, together with the loss of fatty matter, imparts to the whole limb an aspect of attenuation, which, however, upon accurate admeasurement is usually found to be much less than was at first supposed. The cause of this condition of the muscles is evidently twofold: want of exercise and perverted nervous action, leading to atrophy of their substance, as well as to the absorption of the subcutaneous and intermuscular fat. It certainly cannot be explained on the assumption of mere inactivity of the muscles, because an effect so conspicuous as this is never known to follow the want of exercise in a sound condition of the muscular fibre. Unfounded, however, as this explanation is, it is the one usually adopted by surgical writers.

Another remarkable circumstance noticeable in this stage of the disease is an elongated state of the limb, connected with the affected joint. So constant is this occurrence that it may, along with several of the other symptoms above described, be considered as pathognomonic. The extent of the elongation is indefinite, though, in general, it may be said to vary from half an inch to an inch and a half. In rare cases it may amount to two inches and even two inches and a half. It is observed both in the erect and in the recumbent posture, but is commonly more conspicuous in the former than in the latter. Various explanations have been offered of this phenomenon, all, at first sight, more or less plausible. In the first place, it has been argued that it is owing to the presence of an unusual quantity of synovial fluid, the product of inflammatory action, by which the head of the thigh-bone is partially pressed out of its socket and the corresponding limb projected beyond the level of the sound one. No one, however, has yet verified this opinion by dissection. That there is an inordinate secretion of synovial liquor in this stage of the malady is highly probable, but that its quantity is generally so great as to cause such a result is hardly a supposable case. We do know that there are frequently large accumulations of this kind in other joints, as the elbow and knee, without producing such an effect. In the second place, the phenomenon has been ascribed to the relaxed condition of the ligaments and muscles of the joint; but of this occurrence, if it really exist, we have no more positive proof than of the influence which has been attributed to the synovial fluid. A third opinion, and, in my judgment, the only correct one, is that the elongation in question is occasioned by the difference in the level of the two hips, that of the affected side being always lower than that of the sound side. Now, a careful examination of the body will not fail to satisfy us that this difference is real, and not imaginary, and, moreover, that it is always in direct proportion to the increase in the length of the limb. Whatever mode of examination be adopted the result will be the same, whether the patient be recumbent or erect. In the latter case, he is necessarily obliged to support himself upon the sound limb, which, for this purpose, he maintains in a state of rigid extension, at the same time throwing the corresponding hip somewhat outward, so as to bring the axis of the trunk on a line with the sound foot, and thus take off its weight from the affected extremity. This, it will be observed, hangs loosely from the pelvis, upon which the thigh is slightly flexed, while the leg is bent on the thigh, and the foot extended on the leg, the knee projecting prominently forward, much beyond the level of the opposite one, and the whole member resting upon the ball of the foot and toes. If this explanation be correct, as my experience warrants me in assuming, then the elongation of the limb, so constantly witnessed in this stage of the disease, is not real but imaginary, not positive but apparent.

I have never witnessed any shortening of the limb in this stage of the complaint, and doubt very much whether it really ever takes place. The symptom has been described as an occasional occurrence, and ingenious hypotheses have been framed to account for it, but as I have seen no instances of it, I do not deem it necessary to detain the reader with any further remarks concerning it.

Finally, there is generally, in this stage of hip-joint disease, a marked depression, or hollow, in the lumbar region, with a slight inclination of this portion of the spine toward the sound side, and an unusual prominence of the belly. The inferior portion of the spinal groove is also more distinct than natural.

In the third stage the nature of the disease is no longer doubtful, whatever it may have been previously. The symptoms are characteristic, being such as denote the extensive and frightful mischief that has been effected within the joint, in its several constituents. Matter now forms, and, by its pressure upon the inflamed structures, greatly aggravates the suffering. The existence of the suppurative process is indicated by an increase of pain; by a sense of throbbing and tension, deep and persistent; by severe swelling of the gluteal region, generally most prominent at the centre of the articulation; by œdema of the subcutaneous cellular tissue; and by a remarkably turgid and enlarged condition of the subcutaneous veins. The affected joint is intolerant of the slightest motion and manipulation, so much so, indeed, that the patient is unable to raise himself up or turn in bed without experiencing the greatest agony. Every attempt to move the limb is attended by similar results. The constitutional disturbance is always in proportion to the local suffering, and violent rigors, followed by high fever and copious sweats, are rarely absent. Sometimes, however, the abscess forms in a quiet and insidious manner, without any of the severe symptoms that usually accompany the suppurative process in this and other varieties of inflammation. As the matter increases in quantity it gradually works its way toward the nearest surface, its approach being denoted by the occurrence of a circumscribed, erysipelatous blush. Here there is generally distinct fluctuation, and the parts, feeling soft and boggy, soon yield at one or more points, followed by the escape of the contents of the sac.

The site at which the matter, when left to itself, obtains a vent, varies in different cases. Most generally it escapes at the gluteal region, either immediately over the joint, or in its immediate vicinity. The other situations at which it is most liable to discharge itself are the upper and back part of the thigh, a short distance below the great trochanter, the superior and external part of the groin, the sacro-sciatic notch, and the upper and inner surface of the thigh. Occasionally it escapes at several points, either simultaneously or successively, leaving thus a number of orifices, leading to a corresponding number of sinuses. These passages are sometimes very long and tortuous, and in old cases they are always lined by a false membrane. Many years ago I saw an instance in which there were nine distinct openings, and very recently another in which there were as many as twelve; two at the upper part of the thigh, and one just below the crest of the ilium, the remainder being scattered over the gluteal region.

Occasionally the matter escapes both externally and internally. In the case of a young gentleman, named Eaton, aged nineteen, whom I attended with the late Dr. Robert Morehead, the hip was pierced with several openings, while at the same time the matter had passed through the bottom of the acetabulum into the pelvis, round the rectum, through a small ulcer by which it had found its way out, for several months, by the anus. In the female it has sometimes escaped by the vagina, and in both sexes by the bladder. A few examples are on record in which it has passed, by a small opening, into the colon. Sometimes, again, as I have myself seen, the fluid lodges in a kind of sac, formed by the iliac bone and the soft parts of the pelvis, just as pus occasionally lodges between the skull and dura mater.

The matter in hip-joint disease is generally more fluid than ordinary pus, and also of a more greenish tinge. In fact, it very closely resembles the contents of a cold abscess, or the pus of a pulmonary cavern. It is often intermixed with small whitish particles, not unlike grains of soft boiled rice, with flakes of lymph, or even small clots of blood, especially when the antecedent inflammation has been unusually severe, or the parts have been roughly handled. More rarely it contains the debris of articular cartilage, ligamentous tissue, or osseous matter, in the form of little granules of a whitish gritty character. Occasionally, though rarely, large pieces of bone are discharged, and a few instances are related in which the greater portion of the head and neck of the thigh-bone escaped. The pus, when long confined, is often very fetid, and it is always so when, in consequence of a perforating ulcer of the bowel, it is intermingled with fecal matter; very frequently, on the other hand, it is entirely free from odor. Received into a vessel, and permitted to stand for a while, it generally separates into two parts, one at the bottom, solid and granular, the other at the top, fluid, and of a pale whey-like, or oleaginous aspect. When an abscess of this kind has once fully emptied itself, the subsequent discharge is often of a gleety character, ichorous, or thin and bloody.

The quantity of matter may be very small, or so abundant as gradually but surely to exhaust the system. I have repeatedly seen cases where, at the first evacuation, upwards of a pint flowed off, and where, subsequently, the discharge amounted daily, for a number of weeks, to several ounces. At times the suppurative action is almost entirely suspended, perhaps for months, when, either suddenly or gradually, it reappears, and becomes as profuse as before. Once established, it has no special limit as to its duration. I have recently examined a case where the discharge has maintained itself, with hardly any interruption, for two years. The hip and thigh are covered with numerous scars, bearing evidence to the repeated formation of abscesses. The patient is a lad, aged fourteen, much stunted in his growth. Whenever the discharge is unusually protracted it may be assumed, as a general rule, that there is serious and obstinate caries of the bony structures of the joint, attended with the occasional escape of gritty substance.

The symptoms which accompany the discharge of matter are such, usually, as are denotive of hectic irritation. The patient, at least for a time, has regular vesperal exacerbations, the face is flushed, the pulse is excited, the sleep is impaired, and during the night the surface is drenched with copious sweats. Rapid emaciation ensues, the strength declines, and the bowels at length become harassed with colliquative diarrhœa. Thus life may be gradually worn out by exhaustion, or, the discharge diminishing, reaction may take place, followed, sometimes even in apparently desperate cases, by ultimate recovery.

Suppuration, however, does not always take place in this stage of the complaint, or, if it do, the matter is either very small in quantity, or it is so soon absorbed as not to produce its characteristic symptoms. This is true, in many cases, even where the greatest ravages have followed the disease, as the complete disappearance of the articular cartilages, the round and cotyloid ligaments, and even a large portion of the head and neck of the thigh-bone.

It is seldom, according to my observation, that abscesses form externally to the joint in this disease without internal articular suppuration. Indeed, the occurrence is rare under any circumstances. During the progress, however, of the articular malady, the structures over and around the joint, participating in the inflammation, become hard and condensed from interstitial deposits, and from the same cause greatly enlarge in every direction.

The changes which the relative position of the affected limb undergo in this stage of the malady are striking and important, and deserving of special consideration. The extremity, now actually, and not merely apparently, shorter than natural, is much attenuated from the wasting of its fatty and muscular tissues, and remarkably disfigured in its appearance, the heel being considerably elevated, and the ball of the foot and toes alone touching the ground when the patient makes an effort to stand. The degree of shortening is variable, and not always by any means in proportion to the destruction of the head and neck of the thigh-bone, the acetabulum, and the connecting ligaments, which forms so prominent a feature of the disease at this period. While in some instances it does not exceed an inch, or, at most, an inch and a quarter, in others it amounts to twice and even thrice that extent. One-third, and sometimes even one-half of this, as I have satisfied myself by careful examination, is generally attributable to the elevation of the pelvis on the affected side. The position of the foot is variable. Sometimes it looks directly forward, but most commonly it inclines inward or outward, the latter direction being by far the more frequent. These differences are unquestionably due to the extent and nature of the ravages experienced by the hip-joint. When the acetabulum has suffered most severely, the foot usually inclines inward, as in dislocation of the thigh-bone upward upon the dorsal surface of the ilium; if, on the contrary, there has been much destruction of the head and neck of the thigh-bone, and the cotyloid cavity is only slightly involved, then the foot is generally everted, as in fracture of the neck of that bone, the external rotatory muscles tending to draw the whole limb in that direction.

The thigh, as a general rule, is flexed upon the pelvis, the angle of flexion varying from the slightest perceptible change to 45°. In most cases it inclines somewhat toward the sound limb, and occasionally, though rarely, it overlaps or crosses it. Sometimes, on the other hand, it stands off widely and most unseemly from its fellow, as in the case of a woman, aged twenty-five, who was under my charge several years ago. In this case, the two knees are habitually upwards of fifteen inches apart; the affected limb sticks out in the most grotesque manner, and the foot, in the erect posture, is at least six inches from the floor. The extremity of the thigh-bone, the head and neck of which appear to have been absorbed, can be plainly felt in the ischiatic notch. Nearly two years have elapsed since the disease first showed itself; a large abscess formed at the inner and upper portion of the thigh, and for a time the suffering was most severe and depressing. At present, there is an absence of all morbid action, a spontaneous cure having apparently taken place.

The thigh, moreover, is always in a painfully rigid state, owing, probably, to two circumstances, the contracted condition of the muscles of the hip and limb, and the formation of adhesions between the remnants of the head and neck of the thigh-bone and the surrounding parts. By taking hold of the knee a slight degree of flexion may, perhaps, be produced, but to abduct the thigh, or to move it backward, is generally impracticable; besides, every effort of the kind is ordinarily attended with excruciating suffering. Owing to the contraction of the hamstring muscles, the leg is commonly flexed on the thigh, and, for the same reason, the extensor muscles usually draw the heel upward toward the leg.

The great trochanter generally lies directly over the acetabulum, or in its immediate vicinity, forming a hard, firm, immovable, or nearly immovable, prominence, the nature of which it is not possible to mistake. In regard to the head and neck of the thigh-bone, they are, as was previously stated, usually completely annihilated, or so much wasted as to exist only in a rudimentary form. Much has been said by writers respecting the dislocation of this bone in this advanced stage of the disease; but generally in so vague a manner as to convey the impression that their statements are either entirely unfounded, or that they are based upon the assertions of others equally unentitled to credence. Few authors seem to have expressed an opinion upon the subject from personal observation. The valuable facts collected by Professor March, of Albany, in the extensive museums of the United States and of Europe, as well as in private practice, prove, most conclusively, that dislocation of the thigh-bone in any direction is exceedingly rare, as a consequence of this affection. A true luxation, such as occurs in the normal state of the parts, is, in fact, impossible, from the very nature of the morbid alterations experienced by the superior extremity of the bone in question,—its head being generally completely destroyed, and its neck in great measure.

Now, during the progress of the disease, this remnant of the neck, which is usually of a rounded conical shape, and frequently not more than three-quarters of an inch in length, if, indeed, so long, ordinarily places itself over the acetabulum, to the margins of which, and to the adjacent parts, it becomes, in the event of recovery, ultimately united. That it is occasionally drawn up beyond this point, especially when there has been complete destruction of the upper border of the acetabulum, backward toward the sciatic notch, forward upon the pubic bone, or downward and forward into the thyroid foramen, does not admit of doubt. Dislocation, however, in most of these directions can take place only in those cases where there has been extensive suppuration with separation or destruction of the soft parts, allowing the superior extremity of the bone to move about and thus seek, as it were, a new position. The upward displacement is, undoubtedly, most frequent, but even this must be extremely rare. In one of the cases related in a subsequent page, the end of the thigh-bone projected above the acetabulum, where it had formed for itself a superficial socket in the iliac bone, admitting of very slight motion. The real cause, then, of shortening in the third stage of tuberculosis of the ileo-femoral articulation is not dislocation, as has been so often asserted, but the destruction, partial or complete, of the head and neck of the thigh-bone, along with a certain degree of elevation of the corresponding hip.

### III.—DIAGNOSIS.

Although the symptoms of this disease are usually well-marked, especially after the lapse of some time, my observation satisfies me that it is extremely liable to be diagnosticated erroneously. The inexperienced practitioner, misled by the seat of the pain, too often contents himself with a most superficial examination, and, taking this as the basis of his therapeutic indications, is very apt to make a wrong application of his remedies, addressing them, perhaps, solely to the knee, which is only sympathetically involved, when they ought to be directed exclusively to the hip, the actual seat of the morbid action. Numerous cases, illustrative of the truth of this remark, have fallen under my observation, and there are, I am sure, few surgeons in extensive practice who have not, like myself, had occasion to lament the great mischief that has thus been entailed. In a malady so grave as this an error of diagnosis may be fraught with the worst consequences both to part and system, eventuating, as it necessarily must, in the loss of precious time; for it but too often happens that, when the true nature of the disease is discovered, all our efforts to arrest its progress are unavailing.

The affections with which this disease is most liable to be confounded, or which may, at least for a time, obscure its diagnosis, are sprains and rheumatism of the ileo-femoral articulation, psoas abscess, purulent collections in the vicinity of the hip and in the upper part of the thigh, and inflammation of the periosteum of the great trochanter.

A sprain, twist, or contusion of the hip-joint is not an unfrequent occurrence, and may, if followed by considerable inflammation, give rise to severe pain and stiffness, seriously weakening the part, if not completely disqualifying it for the performance of its functions. The consequence is that the patient, in attempting to walk, raises the hip of the affected side and relaxes the corresponding limb, by bending the knee and retracting the heel, very much as in the earlier stages of tuberculosis. The muscles, also, by degrees become flabby and attenuated, and there is a sensible diminution of the temperature of the cutaneous surface. The gluteo-femoral crease is in time effaced, and even the general health may suffer. The signs of distinction are, the history of the case, the absence of pain in the knee, the greater latitude of motion, the absence, in general, of constitutional disturbance, and, lastly, the fact that the foot, although everted, is usually easily rotated on its axis, whereas, in strumous disease of the hip-joint, it is commonly pretty firmly fixed.

Rheumatism of the hip-joint, chronic and subacute, is generally caused by cold, or by sudden suppression of the cutaneous perspiration. It is seated principally in the ligamentous and synovial structures, the cartilaginous and osseous being seldom involved, except in very severe and protracted cases. The pain, which runs down the front of the thigh, is dull, heavy, or aching; the gait is limping, the pelvis is higher on the affected side than on the sound, and the limb exhibits, in the main, the same attitude as in lameness from sprains and contusions, with this peculiarity, that the foot is always strongly everted, while in the former case it is generally inclined inward. The patient in the morning complains of stiffness in the hip, which usually diminishes very sensibly after exercise, but is sure to return in the evening if there has been much exertion or fatigue. The muscles of the thigh are attenuated, but more firm than in tuberculosis, while those of the leg often retain their normal bulk; the gluteo-femoral fold is effaced; the limb, owing to the obliquity of the pelvis, is shorter, often from one to two inches, than natural; the great trochanter is uncommonly prominent; and a creaking noise is generally heard if the head of the thigh-bone be moved forcibly upon the acetabulum. Now, although these phenomena bear a very close resemblance to those of strumous disease of this articulation, yet the absence of severe suffering at night, and at all times at the knee, the marked relief afforded by gentle exercise, the trifling annoyance from pressure, percussion, and motion, even when carefully performed, and the rare occurrence of rheumatism in children, together with the frequent coexistence of this disease in other parts of the body, will generally be found sufficient to prove that the affection is not tubercular.

It is not often that psoas abscess can be mistaken for tubercular disease of the hip-joint; for, although the matter which is poured out in its latter stages, occasionally points to the outside of the groin, or at the upper and inner part of the thigh, there is always the most marked difference in the character of the two swellings, to say nothing of other symptoms. In psoas abscess the tumor is usually situated above Poupart's ligament, while in hip-joint disease it is commonly below; in the former, it always sensibly diminishes and sometimes even entirely disappears under pressure, or when the patient lies down, but quickly reappears when the pressure is removed, or when the patient raises himself up; in the latter, on the contrary, it never changes its position, or if it do, it is in consequence solely of the force of ulceration, absorption, and gravitation; in psoas abscess the swelling receives a distinct impulse in coughing, laughing, and crying, which is not the case in tuberculosis of the hip-joint.

Again, in psoas abscess, the principal pain is in the loins; it is fixed there, and is always greatly increased by the erect posture, as well as by every attempt to extend the corresponding limb. In hip-joint disease, the pain is most severe in the knee, or in the knee and hip. In psoas abscess there is, at no period, any change in the position of the great trochanter, nor any alteration in the length of the limb; in hip-joint disease, on the contrary, especially in its more advanced stages, these are prominent symtoms. Finally, psoas abscess occurs nearly always after puberty, whereas the other affection is most common in early childhood.

Sometimes large deposits of pus take place in the cellular tissue of the nates, or beneath the gluteal muscles, and, forming a prominent tumor in the direction of the ileo-femoral articulation, may thus simulate abscess of the hip-joint from tuberculosis. These accumulations are commonly the result of external injury, or of a phlegmonous, rheumatic, or erysipelatous state of the system, and are, therefore, in general easily distinguished by their history, by the rapidity of their progress, by the severity of the local distress, and by the comparatively prompt recovery of the parts after the evacuation of their contents. Cold abscesses of the nates, besides being exceedingly infrequent, exhibit none of the diagnostic signs of articular disease, especially such as pain in the knee, or pain in the hip-joint upon rotating the thigh, so characteristic of the latter malady. It is only when they depend upon caries of the innominate bone that the distinction would be likely to be attended with difficulty, and in this case a thorough exploration with the probe would probably furnish the requisite light.

Finally, diagnostic embarrassment, to an annoying extent, occasionally arises from periostitis of the great trochanter, in persons of a rheumatic or gouty habit of body. The fibrous membrane of this portion of the femur becomes exquisitely painful and tender to the touch, under the slightest motion and percussion, and the disease, extending above the neck of the bone and capsular ligament of the joint, causes distress and difficulty in walking, with elevation of the corresponding side of the pelvis, similar to what is seen in coxalgia. The soft parts around are swollen and puffy, giving the hip an increased breadth and thickness; by and by, suppuration takes place, sinuses form, and small portions of the bone separate and come away. Unless the case be well managed the joint becomes stiff, and the patient does not regain his health for a long time. The signs of distinction are the persistence of the gluteo-femoral crease, the coexistence of rheumatism or gout in other structures or regions, and the fact that the disease usually occurs later in life than coxalgia.

But it is chiefly in the very early stages of this affection that erroneous views of its diagnosis are liable to be formed; when it is fully established the phenomena are generally too well marked to be mistaken. It has been seen that the very first symptom, in every case, is pain in the knee; so uniform and constant, indeed, is this occurrence that it must be regarded as pathognomonic, and yet, as was previously stated, it rarely happens that it is referred to its true source. Instead of being considered as an expression of disease of the hip-joint, it is too often regarded merely as an effect of neuralgia, rheumatism, or injury of the knee, to which, accordingly, the treatment is exclusively directed. Its great value, as a diagnostic, is totally overlooked, and thus the disease is allowed to progress, at the only time almost when it admits of prompt and radical cure.

In order to avoid this serious and too common mistake, a most thorough examination should be made in every case presenting the slightest suspicion of the existence of tuberculosis of the hip-joint. The very fact that there is pain in the knee, severe in degree, and of frequent recurrence, should of itself excite the alarm of the surgeon; but especially should he be on his guard if, added to this, there is a limping in the gait, an increase of suffering after slight exercise, and disturbed sleep at night. If the diagnosis is obscure, the examination must be repeated, again and again, until it is perfectly cleared up. To conduct the investigation properly the patient must be completely stripped, and viewed both behind and in front, as he stands on the floor. If there be any flattening of the nates, unusual prominence of the trochanter, or change in the gluteo-femoral fold, it will be sure to be detected, and so, also, if there be any alteration in the attitude, size, or length of the corresponding limb. If the patient be now requested to walk, the amount of limping will be discovered, as well as the manner in which he raises and moves the leg and foot. To complete the investigation, the patient is now stretched out on the floor, or on a hard lounge, with a view of ascertaining the amount of suffering produced by rotating the head of the thigh-bone upon the acetabulum, and also by bringing these parts forcibly into contact with each other by percussing the knee, the leg being flexed, or the sole of the foot opposite the ankle, the foot being bent on the leg. The patient being next turned upon his abdomen, the hip is thoroughly examined, first, with reference to the condition of its soft parts, and, secondly, with relation to the sensibility of the component structures of the joint; finally, if there be any obliquity of the pelvis it may be easily observed both in the erect and in the recumbent posture; and any change in the length of the affected limb may be determined by extending a piece of tape, or other suitable band, from the antero-superior spine of the ilium to the inner side of the lower extremity of the patella. The difference in the length of the measure on the two sides will give the difference in the length of the thighs, or the distance between the hip and knee joints. The use of chloroform will often be of great service in conducting the movements of the limb while the patient is recumbent, especially when the parts are very painful and intolerant of manipulation.

### IV.—MORBID ANATOMY AND PATHOLOGY.

The morbid changes induced by this disease vary, as might be supposed, according to the different stages of its progress. As it never proves fatal in its incipiency, all that we know of these changes at this period has been learned accidentally, by examining the bodies of those who have died of other maladies. Enough, however, has been ascertained to show that they do not differ materially, if any, from those of ordinary inflammation. The synovial membrane, which is commonly first implicated in the morbid action, affords evidence of slight vascularity—a few delicate, straggling vessels, loaded with blood, being observable upon its surface, and, in most cases, it is somewhat opake and softened, not uniformly, but at certain points. An appearance of thickening is often imparted to it, from a deposition of lymph, which being poured out, even quite freely in the disease, soon assumes a pulpy consistence and a pale-yellowish color, though occasionally it verges upon greenish. Sometimes it is shreddy, tomentose, or filamentous. The articular cartilage, if seriously involved in the inflammation, is of a dull whitish, or slightly grayish aspect, and somewhat thickened, softened, and partially separated from its osseous connections. The cancellated structure of the bones is abnormally vascular, light, porous, and humid, being at the same time easily broken and cut. Not unfrequently its cells are distended with yellowish tubercular matter, of a semi-solid caseous consistence; or, this substance presents itself in the form of distinct masses, free or encysted, and, perhaps, not larger than a millet-seed. However this may be, these changes are always more conspicuous in the head and neck of the thigh-bone than in the innominate bone, which is seldom seriously affected at this stage of the disease. The round ligament usually suffers early, being abnormally red, tumefied, and softened. The synovial fluid is generally increased in quantity, but rarely to any considerable extent.

As the malady advances, the alterations above described become more distinctly defined; the disorganizing process being now in full play, its devastating effects are plainly visible in every portion of the joint. The lymph gradually increases in quantity, and is often intermixed with a little sero-purulent matter, or thick greenish-looking pus. The synovial membrane is partially destroyed, and what remains is of an opake, muddy, and ragged appearance. The cartilage is ulcerated, pulpified, discolored, perforated, and almost completely detached. The bony structure is very red, soft, carious, rough, and easily crumbled. The round ligament is in a great degree destroyed, and the capsular ligament, as well as the cotyloid, exhibits well-marked signs of inflammation, being loose and spongy at one point, attenuated at another, and perhaps thickened or hypertrophied at a third.

The disease having reached its acme, the structures of the joint are completely subverted, with hardly any traces of their original appearances. Pus is now usually seen, often, indeed, in large quantity, with all the qualities of strumous matter. This, however, is not always the case; for, at times, it is thick and pultaceous, caseous, ichorous, or sero-sanguinolent. In one instance, observed not long ago, I found it very thin, and almost black, from the effects evidently of the necrosed condition of the hip-bone. The synovial membrane, the round ligament, the articular cartilages, and the head and neck of the thigh-bone, with the margins, and frequently, also, the bottom of the acetabulum, are partially destroyed, or even completely annihilated. In the more severe cases the cotyloid, the transverse, and even the capsular ligament are entirely absorbed, the surrounding parts are extensively separated by the ulcerative and suppurative processes, numerous fistulous openings exist, and the gluteal muscles are transformed into dense, firm bodies, of a pale-reddish, yellowish, or whitish color. Cases are not wanting, especially when the disease is of long standing, in which these muscles undergo the fatty degeneration. Occasionally both trochanters are absorbed; or there is extensive caries of the innominate bone; or the head and neck of the thigh-bone are necrosed; or the joint contains numerous fragments of bone and cartilage; or the bottom of the acetabulum being perforated, the matter extends into the pelvis, and passes off by the rectum. In children, prior to the completion of the ossific process, the hip-bone has been found separated, at the acetabulum, into its three primitive pieces.

The head and neck of the thigh-bone being generally absorbed, the remnant of the superior extremity of this bone usually lies across the acetabulum, or in its immediate vicinity. Occasionally it is drawn up a little beyond this cavity, against the surface of the ilium, resting in a slight depression, which, however, bears only a very faint resemblance to a new socket. A few instances, and but a few, have been observed, in which it was found, on dissection, forced backward into the sciatic notch, forward upon the pubic bone, or downward into the thyroid foramen. When the disease has expended itself mainly upon the acetabulum, the head of the thigh-bone may remain in its original position; or it may even pass, as, in fact, it has been known to do in several cases, into the pelvis.

If death takes place after a process of recovery has been set up, the acetabulum will be found filled by a white, fibrous, organized substance, the extremity of the thigh-bone being anchylosed, or firmly attached by new matter to the surrounding structures. It is very rare for a new socket to be formed; yet that this is not impossible is proved by dissection, as well as by specimens in different museums. In time, the artificial joint may admit of considerable motion, as is seen in persons who have recovered from the disease, attended with great shortening of the limb; but, in general, the motion is extremely restricted. Sometimes an imperfect ligament is formed round the bony knob above described, and the surface of this bony knob may even become slightly tipped with cartilage. Finally, osseous growths, short, irregular, and friable, occasionally make their appearance upon the thigh-bone, and also upon the innominate bone, in the vicinity of the former disease.

The bodies of those who die of strumous disease of the hip-joint, or while laboring under this affection, usually exhibit serious pathological changes in some of the internal organs. These changes appear to be the direct result of the tubercular cachexy, which is generally so well marked in the latter stages of the local malady, and they exist in various forms and degrees in different structures. The most common are tubercular deposits and dropsical effusions, which are rarely entirely absent in any case, especially if of long standing.

Tubercles of the lungs are very common; they often exist in great numbers, especially in the summits of these organs, and they always exhibit the same characters as in ordinary phthisis. Cavities sometimes form, but death usually occurs before they attain any considerable magnitude. The bronchial ganglions commonly participate in the pulmonary disease, being enlarged and tuberculized. Occasionally extensive adhesions are found between the lung and costal pleura, with or without serous and other effusions. The heart is seldom affected.

The peritoneum is sometimes extensively tuberculized, and considerable quantities of water are often found in its cavity. In children, the lymphatic ganglions of the pelvis and mesentery are apt to suffer from strumous deposits, and similar changes are occasionally witnessed in the spleen. The liver is often cirrhosed and hypertrophied. Now and then the glands of Peyer suffer. In one of the cases mentioned below, they were pretty extensively ulcerated. The pancreas, stomach, and genito-urinary organs are usually sound. The rectum, and even the colon, the vagina, and the bladder, are sometimes found perforated, the matter passing directly from the hip-joint through the abnormal aperture. The blood is generally very thin, and deficient in fibrin and coloring matter. The lower extremities, and even the hands, face, and genital organs are, at times, anasarcous, especially when the system has been worn out by tubercular disease of different parts of the body.

Tuberculosis of the hip-joint, as the name correctly implies, is essentially a scrofulous disease, bearing the same relation to the structures of that articulation that phthisis bears to the lungs; in other words, it is, to use a common but sufficiently expressive phrase, like scrofulous complaints in general, merely a local symptom of a constitutional disorder. Take away this constitutional vice, this strumous cachexy, and in either case no local disease can arise. That this is not a mere assumption, but an indisputable fact, is abundantly established by the symptoms and progress of the local affection, and by the appearances revealed on dissection of those who die of it.

Tubercular disease of an organ does not necessarily suppose in that organ the existence of tubercular deposits. In strumous corneitis, for example, we find nothing of the kind, and yet no one, at all familiar with the character of that malady, would deny it such a parentage. In certain diseases of the skin there is undoubtedly scrofulous action, without, so far as can be determined, any secretion of tubercular matter. When the disease begins in the synovial membrane of the hip-joint, it probably deports itself in the same manner as when it invades the tunics of the eye; and the same thing, there is reason to conclude, occurs when it takes its rise in the cartilaginous tissues. When, on the contrary, it commences in the osseous structures, whether in the thigh-bone or in the innominate bone, there may, and no doubt often is, a genuine deposit of this kind, such as is so frequently met with in the short bones, as those of the spine and foot, and also in the articular extremities of some of the long bones. Such, then, it is believed, is the true pathology of this affection; but to enter into any further discussion here would be to describe the pathology of strumous affections in general, for which this is not the proper occasion.

### VI.—CASES OF HIP-JOINT DISEASE.

The following cases and dissections are introduced here as further illustrations of the progress, pathology, and complications of this affection.

CASE I.—Eliza Kennedy, aged nine years and a half, the oldest of four children, of fair complexion, with light hair and eyes, and of a naturally delicate, strumous constitution, became the subject of tubercular disease of the left hip-joint in April, 1841, soon after a severe attack of measles. For the first few months she suffered severely from pain in the knee and inner part of the thigh; but about this period it left these points, and fixed itself at the seat of the morbid action, shifting its place, however, occasionally, especially after exercise, from which her physician did not restrain her. The gluteal region was much swollen, and the limb gradually became stiff and useless. Thirteen months had elapsed before I saw the case. Suppuration had already taken place, and several sinuses existed in the neighborhood of the ileo-femoral articulation, discharging a considerable quantity of thin gleety-looking matter. The limb was inverted, much shortened, and in close contact with the sound one; the thigh being, at the same time, a good deal flexed upon the pelvis. The child was hectic, greatly emaciated, racked with pain, and harassed with diarrhœa and night-sweats. In a few months a hacking cough set in, which rapidly increased in severity, and, together with other symptoms, only too clearly indicated the existence of confirmed phthisis. Death occurred nearly two years after the first signs of hip-joint disease, preceded by slight ascites, anasarca of the lower extremities, and the formation of new sinuses in the gluteal region.

Both lungs were crowded with tubercles, especially at their summits; most of them were in a crude state, but several were quite soft, and, on the right side, a cavity existed capable of containing a pigeon's egg. Extensive adhesions were found between the costal and pulmonary pleuræ. The bronchial ganglions were enlarged, but not tuberculized. The heart was normal. The peritoneal cavity contained nearly half a gallon of limpid serum. The spleen and mesenteric ganglions were crowded with tubercular matter, mostly in a crude state. The stomach, bowels, liver, pancreas, and genito-urinary organs were sound.

The gluteal region was unnaturally hard and prominent, and the seat of four sinuses, three of which communicated with the diseased joint, while the other, situated just below the antero-superior spine of the ilium, led to the interior of the pelvis. By pressure, a small quantity of thin gleety fluid could be forced from these openings. The principal prominence of the hip was formed by the great trochanter, which lay immediately beneath the skin, and much nearer to the sacro-sciatic notch and the tuberosity of the ischium, than in the normal state. The head and entire neck of the thigh-bone, except a small tubercle, hardly half an inch long, and of a rounded shape, had disappeared by absorption. This remnant, which was quite rough and considerably softened, closely embraced the acetabulum, and was directed backward toward the sacro-sciatic notch, from which it was distant about three-quarters of an inch. The shaft of the thigh-bone was strongly flexed upon the pelvis, and inclined over toward the opposite side, thereby throwing the trochanter prominently outward and upward. The acetabulum had lost its cup-like appearance, its margins having been removed by absorption, leaving thus a shallow elongated depression, closely embraced, as just stated, by the remains of the neck of the thigh-bone. The capsular ligament was entirely destroyed, except a small portion at the outer and upper part of the joint, which was much thickened and otherwise altered. There was no dislocation, properly so termed, though the two articulating surfaces had undergone great changes in their relative position. Complete anchylosis existed. The gluteal muscles were remarkably pale, and so much wasted as to render their fibres very indistinct. All the soft structures about the joint were excessively indurated, from the deposition of plastic matter. Running along the inner surface of the innominate bone, in a transverse direction, was a long sinus, which opened, as already seen, just below the anterior spine of the ilium.

CASE II.—James Franklin, a negro, aged ten years and a half, the third of eight children, was attacked with symptoms of hip-joint disease early in December, 1853. His health, up to that time, had always been delicate, although he had never been confined to his bed. None of the members of the family had ever suffered from scrofula in any form. Among the earlier symptoms complained of were a sense of uneasiness in the corresponding knee, and some degree of stiffness in the hip, causing a slight halt in the gait. As the affection advanced the lameness increased, and the boy experienced severe pain in the knee, usually aggravated during the night and during changes of the weather. Superadded to these symptoms were frequent startings of the limb during sleep, causing the patient to wake, and even to scream out. On such occasions he was in the habit, for several months, of jumping up in bed in a species of fright or partial delirium, some time being generally required to rouse him sufficiently to make him comprehend his situation. By-and-by he was able to exercise only on crutches; his general health began to decline, and he became sensibly emaciated. When I first saw him, early in November, 1854, he was hardly able to walk or stand, on account of the stiffness of the hip-joint; and it was evident that his general health had greatly suffered. On examining him I found there was great flattening of the nates of the left side, with complete effacement of the groove between the thigh and hip, and elevation of the corresponding iliac crest; the knee was greatly flexed, and, on holding the boy up in the erect position, it projected several inches beyond the sound one, the heel being, at the same time, raised more than two inches from the floor, and the toes inverted. Some motion existed at the hip in the direction of flexion, but the thigh could neither be extended nor drawn away from its fellow. The abdomen was considerably enlarged, but there was an entire absence of disease of the glands of the neck.

A week after I first saw the boy, I applied the actual cautery, with a view of establishing a large issue. The eschar separated about the usual time, and the resulting sore seemed to be productive of some benefit, as was evinced by the diminution of the pain, and the improvement in his appetite and sleep, the former of which became quite voracious. About the middle of December the tumidity of the abdomen began sensibly to increase; he now voided but little urine, and he suffered under great embarrassment of breathing, accompanied with frequent and severe paroxysms of coughing. It was evident that he was laboring under ascites and serious disease of the lungs. Growing gradually worse, he expired on the 11th of February, 1855, having for the last fortnight labored under excessive œdema of the legs and scrotum, and been unable to lie down for a single moment on account of his dyspnœa.

The body was examined the day after death, with the assistance of Dr. William H. Lyle.

The abdomen contained about three quarts of limpid serum; the peritoneum, especially the pelvic division, was studded with an immense number of incipient miliary tubercles; the glands of Peyer were ulcerated over a considerable surface, and immediately over each ulcer the serous covering was studded with well-developed tubercles. The spleen was filled with similar bodies, but they were much larger, and evidently in a much more advanced state. The mesenteric ganglions formed a large indurated mass, each gland being filled with tubercular matter, which, in some of them, had advanced toward softening. The pelvic lymphatic ganglions were likewise enlarged and tuberculized. The stomach, liver, large bowel, and genito-urinary organs were healthy. Both lungs had contracted extensive adhesions, evidently of long standing, to the walls of the chest; on the right side they were formed by a false membrane at least one-third of an inch in thickness, and presenting here and there tubercular deposits. The right lung was much softened, hepatized, and extensively tuberculized. Upwards of eight ounces of serum was found on this side of the chest. The left lung was nearly sound, but contained some tubercles at its summit. The heart and large vessels were normal. The bronchial ganglions were enlarged and tuberculized.

The gluteal muscles were pale, indurated, much wasted, and closely matted together. The round ligament, and the whole of the capsular, except the inner and inferior portion, were destroyed; what remained was thickened, indurated, and ragged. The upper extremity of the head of the thigh-bone was absorbed, leaving a rough, scabrous surface, partially incrusted with lymph. The remainder of the head was much softened and covered with cartilage, which, however, was entirely loose over a large space on a line with the great trochanter, as well as thickened and softened in its texture. The neck of the bone was hardly half an inch in length, and completely horizontal in its direction. The thigh-bone was strongly inclined inward, and flexed upon the pelvis. The hip was rendered extremely prominent by the projection of the great trochanter. The bottom of the acetabulum was thickly incrusted with organized lymph, and perforated at its iliac side; its edges, which were still covered with fibro-cartilage, being softened and partially absorbed. Directly over the opening, on the pelvic aspect of the innominate bone, lay an enlarged lymphatic ganglion. The head of the thigh-bone still retained its place in its socket, though it was drawn up more than half an inch beyond its natural situation. The two articulating surfaces were closely united by organized and tolerably firm lymph.

CASE III.—The subject of this case was Harriet, a negress, aged twelve years. She had always been healthy, and no member of her family had ever had scrofula. A short time before she was attacked with hip-joint disease she became affected with inflammation of the eyes, which the physician in attendance pronounced to be of a strumous nature. It soon yielded to treatment.

About two years and a half ago, she began to complain of a severe pain in the left hip and knee, particularly the latter, which was supposed to be of a rheumatic character, and was treated accordingly. No amendment, however, followed; instead of this, the symptoms steadily progressed, the local distress increasing in violence, and the limb becoming so much contracted at the knee as to cause considerable lameness. The pain, at length, after some months, entirely ceased, but the difficulty of walking became so great that she was unable to move about without the aid of crutches. The general health, meanwhile, was not materially impaired; she was rather thin, however, and her sleep was occasionally interrupted by spasmodic twitches of the limb. Such was her condition at my first visit, in December, 1854. Upon examination, I found the characteristic flattening of the nates, the effacement of the gluteo-femoral groove, and the elevation of the iliac crest, with very marked shortening of the limb, which was bent at the knee, and inverted at the toes. Every attempt to draw it away from the opposite one was attended with severe pain, and similar effects were produced by percussion of the knee and foot. Pressure over the hip-joint caused considerable uneasiness, but no pain, properly so-called. The child was much emaciated, sleeping badly at night, and having but little appetite. She was unable to stand, except when assisted, and she experienced frequent attacks of pain at night.

The application of the actual cautery, a few days after my first visit, had the effect of relieving her pain, and of improving her general condition. After a few weeks, however, she became worse, being seized with a troublesome diarrhœa, which continued to harass her until she died. About three weeks before this event, she became affected with a severe cough and some dyspnœa, and there was a return of pain in the hip with all its original intensity. She now lay constantly on her right side; had fever most of the time, especially at night; the emaciation rapidly increased, and she died, completely exhausted, on the 18th of February, 1855, less than two months and a half after I took charge of the case. The immediate cause of her death was evidently pneumonia and diarrhœa.

About a pint of serum was contained in the peritoneal cavity. The mesenteric glands were considerably enlarged, and pervaded with tubercular matter. The alimentary canal, the liver, spleen, pancreas, kidneys, and bladder were healthy. Both lungs exhibited evidence of pneumonia, but the right in a greater degree than the left, and the former also contained, especially at its summit, a number of miliary tubercles, for the most part in a solid state.

The gluteal muscles were much reduced in size, pale, indurated, and apparently in a fatty condition. The round ligament and all of the capsular, except the lower posterior portion of the latter, had disappeared. The acetabulum was unusually deep, from the absorption of its bony walls, which were very rough, and deprived of cartilage. At its centre it had the appearance of giving way; and at its supero-posterior part, opposite the sciatic notch, was an opening large enough to receive the end of the thumb, caused by the detachment of a piece of bone. The margins of the acetabulum retained their natural sharpness and covering. The pubic bone was ulcerated and softened at its cotyloid extremity. The neck of the thigh-bone was abnormally short and horizontal, and encircled by the remains of the capsular ligament. About one-half of the head was gone, the portion which was left having a rough, scabrous surface, the exterior of which was covered with cartilage in a state of inflammation and partial softening.

CASE IV.—Timothy Connelly, twenty-six years old, a day-laborer, of intemperate habits, and a native of Ireland, was admitted into the Louisville Marine Hospital, on the 3d of September, 1854, on account of disease of the left hip-joint, first perceived six months previously. The earliest symptom in the case was a pain in the joint and its immediate vicinity, accompanied with slight swelling, which gradually increasing in severity, at length extended down the limb as low as the middle of the thigh. At the time of his entrance he was unable to use the limb in standing or walking, and he complained of severe pain in the hip, knee, and back. The thigh was somewhat flexed on the pelvis, the heel was raised upwards of two inches from the ground, and the foot was inverted. During the last two weeks the swelling of the thigh had much increased, and the parts were now quite tender on pressure, with a distinct sense of fluctuation. A free incision being made, a large quantity of dark-colored and offensive matter was discharged, evidently scrofulous in its character. A tent was introduced to promote the escape of pus, which continued for several weeks. The man was placed on tonics and alteratives, under which he seemed to improve somewhat. Sometime after, the opening in the abscess being permitted to close, matter again accumulated in the sac, requiring another puncture. This was made on the 28th of November, before the medical class at the Hospital, and gave vent to about sixteen ounces of ill-elaborated and fetid pus. A tent was again inserted, and the patient was put upon the use of tonics with a nutritious diet. But little improvement followed, debility being a prominent symptom, although there was seldom any pain in the hip or knee, except when he attempted to walk. On the 10th of January, 1855, the man was again brought before the class, when I laid open a long sinus, extending along the antero-external part of the thigh, toward the interval between the great trochanter and the anterior spinous process of the ilium, the incision being at least eight inches in length. The same treatment was continued, with the addition of a compress and bandage to the affected limb.

On the 1st of March, when my tour expired, the wound was still unclosed, discharging daily a small quantity of unhealthy-looking pus, and manifesting but little disposition to heal The patient was gradually growing weaker, and now complained of a great deal of pain in the hip-joint and lumbar region. Cough, and a sense of constriction above the chest, accompanied with a dull pain, were a source of frequent annoyance to him. The progressive emaciation and debility, with other corroborative symptoms, led to the suspicion that the original affection was now complicated with pulmonary phthisis, and the suspicion was fully verified by a careful examination. He continued gradually to decline, and died, completely exhausted, on the 3d of May, 1855.

The body being inspected two hours after death, the legs and feet were found to be quite œdematous; the left foot and knee were much inverted; and the limb was an inch and three-quarters shorter than the sound one. The peritoneal cavity contained upwards of a quart of water, but all the abdominal viscera were sound. The kidneys, supra-renal capsules, bladder, and prostate gland were free from disease. The lungs contained crude disseminated tubercles at their summits,—a few verging on softening. Similar deposits were found in the bronchial lymphatic ganglions. The pleura and the heart were normal. The brain was not examined.

The acetabulum was completely denuded of cartilage, very rough, and of an unnaturally darkish color; its edges being beveled off, it presented an elongated, ovoidal shape, being three inches in height and two inches and a half in breadth. The capsular and round ligaments were entirely destroyed, but the transverse was apparently unaffected. Extending inward, along the ramus of the pubic bone, was a large sinus, filled with a black, offensive fluid, which also existed, in considerable quantity, in the joint itself. The head and the whole of the neck of the thigh-bone, except a short, thick, rough stump, had disappeared. The great trochanter rested against the upper edge of the cotyloid cavity, the upper extremity of the latter being received into a small depression of the former. From the flexed position of the shaft of the bone, the trochanter lay across the acetabulum in an oblique direction, and consequently much nearer both to the spine of the ilium and the sacro-sciatic notch than in the natural state. The articulating surfaces were firmly united by plastic matter, and, although their relative position was greatly altered, there was no dislocation. The gluteal muscles were much attenuated, very pale, indurated, and matted together. Some of their fibres had evidently undergone the fatty degeneration.

Case V.—Thomas Farrell, Irishman, aged twenty-three, laborer, with black hair and eyes, pale complexion, and delicate constitution, was admitted into the Louisville Marine Hospital, February 17th, 1852, for what, at the time, was supposed to be typhoid fever. His principal symptoms were cough, muco-purulent expectoration, and hectic irritation, under which, becoming gradually exhausted, he expired on the 16th of March. He had had hip-joint disease some years previously, but had so far recovered from it as to have been induced to believe that a spontaneous cure had taken place.

The dissection was made several hours after death. The body was sallow, and much emaciated The right lower extremity was three inches shorter than the left, the toes were much inverted, and the sole of the foot rested against the instep of the sound one. The knee was also turned inward, and three inches above the opposite one, but not in advance of it. The great trochanter was extremely prominent, and distant three inches and a quarter from the antero-superior spinous process of the ilium. Four old scars existed in the groin and round the great trochanter. The gluteal muscles were much wasted, and transformed almost completely into a pale, whitish, fatty, and fibrous substance. The head and neck of the thigh-bone had been absorbed, being represented by a hard, ivory-like, rough, scabrous knob, mounted some distance above the acetabulum upon the iliac bone, where it had formed a superficial socket, extending into the original cavity, which was filled up by osseous matter. The new joint thus formed, however, hardly admitted of any motion, and some force was required to break up the adhesions between the contiguous surfaces. The capsular ligament immediately round the new joint was very dense, and nearly three lines in thickness. All the rotator muscles of the limb were pale and atrophied, while the psoas and iliac muscles were completely transformed into fibrous tissue.

Both lungs were crowded with gray granulations, which were particularly abundant in the summits of these organs. In none of them had the softening process commenced. Both lungs were of a dark-purple color, heavy, and much engorged with blood. Ancient adhesions existed on both sides, but more extensively on the left than the right. The bronchial tubes and the lymphatic ganglions were sound; as also the heart.

The spleen, liver, and kidneys, especially the first, contained an immense number of tubercles, discreet, small, rounded, and solid. Of those in the liver, which was of the normal bulk, many were tinged with bile. The spleen was considerably hypertrophied, and the kidneys, particularly the left, were of unnatural size. The ureters, bladder, and prostate gland were healthy. Many of the mesenteric ganglions were enlarged and tuberculized. The stomach, bowels, and pancreas were natural.

CASE VI.—The following case is subjoined as exhibiting the state of the part and system in a somewhat advanced stage of the disease:—

Mary Ziegler, German, aged six years, light hair, eyes, and complexion, was seized with symptoms of hip-joint disease, in October, 1854. In the month of June previously she had a severe attack of cholera, which left her, until late in the season, in a very feeble and emaciated condition. Up to this time she had always enjoyed good health. The affection of the hip-joint came on without any assignable cause. During the first few months the child suffered excruciatingly both day and night, the pain shifting about from the hip to the knee, and from these parts to the abdomen and the lower dorsal vertebræ, which, as was very apparent from their posterior curvature, must also have been the seat of tuberculosis. The upper and inner portion of the thigh was likewise a frequent seat of severe pain. In consequence of the violence of her suffering the little girl was deprived of sleep and appetite, and became again rapidly and greatly emaciated. Toward the spring of 1855 she began to improve for a short time, and the parents flattered themselves that she would gradually recover. These hopes, however, proved delusive, for after a few months all her distress returned, and late in the season, that is, sometime in November, a large abscess formed in the upper and forepart of the thigh, just below the groin, and soon breaking, discharged upwards of a pint of pus. This afforded great relief, for previously to this event, for several months, she endured excessive pain. This opening still existed, together with another on the inside of the thigh, near the groin, and one on its posterior surface, about two inches below the great trochanter. The discharge from them was very slight.

The present condition of the child, March 17th, 1856, is as follows:—She is in great degree free from pain, rests well at night, sits up nearly constantly in the day, and has a pretty good appetite, with a reasonable share of flesh and strength. She has still some diarrhœa, under which she has labored, more or less, ever since the commencement of her illness. The right hip is much larger than the left, as well as more flat, and considerably elongated transversely. The crease between the thigh and buttock is effaced, and the swelling extends, in a tapering manner, as far down as the upper third of the thigh. The surface feels hard, and has a perfectly smooth appearance, except at the site of the trochanter, where it is a little rough. The trochanter itself is quite prominent and firmly fixed in its new position. The thigh is in close contact with its fellow, and considerably flexed on the pelvis; the knee is in advance of and above the other; the leg is flexed on the thigh, and the foot is extended on the leg. When the child stands up the foot looks directly forward, but by taking hold of the ankle and moving it, it can be slightly inverted or everted. When in this position she supports herself on the ball of the toes, the heel being elevated upwards of two inches from the floor. The spine, at the lumbar region, inclines a little over toward the affected side, and the iliac crest on that side is somewhat higher than that of the left. The projecting dorsal vertebræ are quite prominent, but free from pain and tenderness. The probability is that the morbid action is suspended, and that a spontaneous cure is taking place.

### VI.—PROGNOSIS.

Tuberculosis of the hip-joint is essentially a chronic disease, which, after having endured for an indefinite period, terminates either in recovery or in death. The recovery may be complete, both as it regards the part and system, or, the local action disappearing, the joint may be left weak and anchylosed, and the general health regain its original vigor; or, as not unfrequently happens, particularly after the process of disorganization has commenced, both the articulation and the constitution may remain in a degraded and crippled condition for a long time, or even during the rest of life. No mortuary statistics of this affection have yet been furnished, and it is, therefore, impossible to state, with any degree of precision, the mean duration of fatal cases, or the relative proportion of deaths to recoveries. My opinion, founded upon numerous observations, is, that the mortality from the disease is slight in almost any event, even where there has been palpable neglect in regard to treatment, medical, surgical, and hygienic. In this view, I believe, most writers fully concur. When death does take place, it rarely happens before the eighteenth month, and very often not until after the second year.

Many circumstances, mostly of an individual nature, conspire to influence the prognosis in this disease. So much, in truth, is this the case, that it is utterly impossible to lay down any definite rules for the guidance of the practitioner. Much will, doubtless, depend, in every instance, upon the state of the constitution, the presence or absence of complications, and, above all, upon the duration of the malady. Age also exercises an important influence. It is a well-established fact that, other things being equal, children will live longer, and also stand a much better chance for recovery, than young adults and middle-aged subjects, in whom, especially the latter, the disease often proceeds with extraordinary rapidity, sometimes ending fatally in a few months. When the constitution is naturally feeble, or when it has been rendered so by the intensity of the local suffering, the probability of an unfavorable termination will be much increased. Imperfect alimentation, however induced, is another source of mischief, both as it respects the part and system. Intercurrent maladies, such as typhoid, intermittent, and eruptive fevers, diarrhœa, dysentery, and erysipelas, often retard recovery, or hasten the fatal crisis. These and other diseases, by establishing a drain upon the system, already exhausted by the local suffering, are, I am satisfied, the principal causes of the mortality in strumous affections of the hip-joint. When the malady proves fatal without such intervention, it will generally be found that death is directly due to the depressing effects of tuberculosis of the lungs, lymphatic ganglions, spleen, or peritoneum, which is so liable to show itself under such circumstances.

Much of the mortality of this disease, as well as most of the bad effects, both temporary and permanent, which it entails upon the affected articulation, results from neglect of appropriate management, prior to the commencement of the disorganizing process. Properly treated at its beginning, it is, at least in the majority of cases, as amenable to our remedial agents as any ordinary chronic inflammation, although not generally so promptly. The morbid action gradually receding, the effused materials are by degrees absorbed, and the parts restored to their normal functions. Arrived at its second stage, hardly any course of medication, however judiciously applied, will completely avert permanent rigidity, although life may not be at all in jeopardy. The morbid impression has already advanced too far to admit of easy recession; a certain amount of organic lesion is present, and the patient may congratulate himself if he ever completely regains the use of his hip. In the third stage, when the osseous, cartilaginous, and other structures are disintegrated and broken down; when, perhaps, the interior of the joint is converted into a large chronic abscess; when the lower extremity is shortened and stiffened, and, finally, when the constitution is worn out by pain and hectic irritation; there will not only inevitably be loss of function, but also danger of loss of life. If, under such circumstances, the patient survive, his recovery will be effected at the expense of much suffering, too often eventuating in premature decay and dissolution.

### VII.—TREATMENT.

In the treatment of this affection, which, if our notions of its pathology be well founded, is, like scrofulous disease in general, merely a local manifestation of a constitutional vice, topical remedies alone will not avail; to prove efficient and truly useful they must be combined with and aided by means addressed to the general system with a view to the improvement both of the solids and fluids. It would be idle, in the present state of the science, to insist upon a course so palpably proper in itself and so long sanctioned by experience. As well might the practitioner expect to be able to cure consumption, or to ameliorate the condition of a person thus affected by the exclusive employment of counter-irritation and other external measures, as to cure tuberculosis of the hip-joint without the aid of constitutional remedies. Again, in treating this disease it should not be forgotten that it consists of different stages, which, although they run into each other by imperceptible gradations, are nevertheless of vast importance in a practical point of view.

Whatever may have been the duration of the malady when the treatment is commenced, the first and most essential element in the management of every case of the kind is repose, not merely of the limb alone, but also of the body,—repose absolute, unconditional, and persistent. Upon this subject there must be no compromise between the patient and his attendant. The contract entered into at their first interview must be observed with the most scrupulous fidelity,—it must be both binding and permanent. The slightest departure from this injunction would in any stage of the complaint be of great detriment to the patient's limb, while in its more advanced stage, when the bones and cartilages are destroyed and matter exists in the joint, it might seriously jeopard his life. Recumbency must be observed for months upon months. Too much stress cannot be placed upon this point, when it is remembered how important it is to keep parts, comparatively insignificant, perfectly at rest when in a state of suffering. In inflammation of the fingers and toes repose and elevation of the affected structures are instinctively sought, and, if possible, maintained until the morbid action has in a great degree subsided and function been restored. Now, if this be necessary in ordinary inflammation and in textures which have no important uses and sympathies, how much more necessary is it when the malady is of a specific nature and the organ involved is so important as that of the hip-joint? These facts are well known to practitioners, but the common people are ignorant of them, and hence they should always be thoroughly explained to the patient and his friends at the very threshold of the treatment.

In order to render the patient as comfortable as possible, and enable him to endure his protracted confinement without detriment or inconvenience, he must be furnished with a suitable bed provided with slats and a firm but elastic mattress. A common trundle-bed, about four feet in width, will answer every purpose, and is in every respect preferable to the common bed, especially if the patient is a child, as he will thus be less liable, if he should roll out, to hurt himself. The sheet should be well secured at the sides that it may not become rumpled, and the pillow should be of medium size, so that, while it affords adequate support to the head and shoulders, no undue weight may be thrown upon the trunk and pelvis. The confinement, if rigidly insisted upon, will not prove irksome; with the aid of toys and other sources of amusement the little patient will soon,—often, indeed, in a few days,—become reconciled to his new mode of life.

The local remedies must be regulated by the progress of the malady and the constitution of the patient. If the disease be in its first stage, if the pain be violent and frequent in its recurrence, and if the general health remain unimpaired, we can scarcely fail to derive special benefit from the application of leeches, or, in their absence, from the use of a large blister. The leeches should be scattered over the affected joint, and after they have dropped off, the flow of blood should be promoted for some time with cloths wrung out of warm water and frequently renewed. Their number must depend upon circumstances, but in general from six to eight will be sufficient for a child from three to six years of age. Sometimes a blister may be applied advantageously within a few days after the leeching, and I much prefer this mode of counter-irritation to the employment of liniments, embrocations, croton oil, tartar emetic ointment, and iodinized lotions, which is always attended with friction, and for that reason often prejudicial to the inflamed structures. By these means, aided by a plain, simple diet, consisting mainly of farinaceous articles, with milk, weak tea, or milk and water, at breakfast and supper, and an occasional purgative of from two to three grains of blue mass with double that quantity of jalap, most of the cases that are brought under the notice of the practitioner may be radically cured in a few months. If fever be present, or if there be decided plethora, as denoted by the state of the pulse and face, the antimonial and saline mixture may be given in such doses and at such intervals as the nature of the case may seem to require. When the pain is so severe as to interrupt sleep, opiates must be used with warm anodyne poultices or fomentations, or, what I have found to be of greater benefit, a lotion composed of three parts of soap liniment and one of acetated tincture of opium with from half a drachm to a drachm of carbonate of potassa to the ounce, applied by means of a fold of flannel, kept constantly wet, and covered with gutta-percha. Cold applications are generally inadmissible both in this and in the other stages of the disease. Where the skin is unusually dry, or the system more than commonly irritable, the warm bath, carefully administered for half an hour at a time, with a Dover's powder at night, is sometimes highly beneficial, but great care must be used in its administration lest the affected joint sustain injury.

Such is an outline of the treatment which, according to my experience, will usually be found most serviceable in the earlier stages of the complaint. There is, however, not a little diversity in regard to the nature of the internal remedies required in different cases. As already stated, there are two distinct classes of patients, the plethoric and the anæmiac, those with an apparently rich blood and those with an impoverished blood, and hence two very opposite courses of treatment are frequently necessary, the former demanding perhaps a certain amount of depletion, while the latter will be most benefited by tonics. I have not met with any cases where I thought the use of the lancet was indicated, and yet I am not prepared to say whether, when the inflammation and pain are very great, in a strong, robust child, soon after the commencement of the malady, venesection, to a small extent, might not be highly beneficial, tending to retard the suppurative crisis, and to prevent the destruction of the osseous and cartilaginous tissues; in general, however, the remedy would be too harsh, and I am satisfied that the morbid action may be sufficiently repressed by the antimonial and saline mixture, in union with a minute quantity of tincture of aconite, assisted perhaps by the application of a few leeches to the seat of the disease. In the anæmiac, a not uncommon class of cases, tonics and stimulants are often required at the very commencement of the disease, consisting either of quinine and iron, or, what is peculiarly valuable under such circumstances, cod-liver oil, in such doses as shall not prove offensive to the stomach. The diet should of course be of a corresponding character, and the patient should, if possible, be induced to use milk-punch, wine, ale, or porter, in order to rebuild the system, and thus enable the affected parts the more effectually to resist the encroachments of the morbid action.

I am not an advocate, as a general rule, for confining the affected limb in splints, as is the custom of many practitioners, with a view of securing more perfect rest of the joint. The patient himself will usually instinctively take care of this. It is only or chiefly when the limb is much out of shape, or in a position in which, if permanently retained, its usefulness would be seriously impaired, that I have found such a proceeding either necessary or desirable. The restraint occasioned by all such contrivances is generally exceedingly irksome to the child, and is often, I am sure, productive of harm rather than of benefit. When the malposition of the extremity is considerable, the first thing to be done, provided there is as yet no great structural lesion, is to rectify this by extension and counter-extension, aided, if necessary, by rotation and abduction, the patient being under the influence of chloroform, and then to apply a suitable apparatus for maintaining the limb in its new relations. The best material is gutta-percha, undressed sole-leather, or trunk-maker's board, soaked in hot water, and adapted to the hip, thigh, and leg, being kept in place by a common roller. When dry, these articles form an admirable case, which may afterwards be padded with cotton to ward off pressure, and which will thus effectually prevent motion not only of the hip but also of the knee and ankle.

The attempts at rectifying the malposition of the limb are occasionally annoyingly counteracted by the contraction of the principal adductor muscles of the thigh, especially the pectineal and short adductor, which are sometimes so much shortened as to draw the extremity nearly across the sound one. When this is the case, the most expeditious plan is to divide these muscles subcutaneously; and the effect of such an operation upon the future welfare of the part and system is often most striking, the pain and spasm being relieved as if by magic, and the limb becoming perfectly docile and manageable.

In the second stage of the affection, the only reliable local remedy, according to my experience, is a large issue, for the purpose of securing a free and permanent discharge of matter. If the case have not been seen before it has reached this crisis, some of the means already mentioned, as leeching and blistering, may be tried; but, unless they are promptly beneficial, no time should be wasted in their employment. The disease is now thoroughly established, and must be met in the most decisive manner, if we would wish to avoid its disorganizing and destructive consequences. The best place for making the issue is the most prominent part of the swelling, which is usually either directly over the joint or in its immediate vicinity. It is reasonable, *a priori*, to suppose, that the nearer the discharge is established to the diseased structures, the more likely will it be to be useful; and this is precisely what experience has shown to be the fact. The place of election, then, is a circumstance of great importance, and should not be overlooked.

The most eligible form of issue is that made with the actual cautery. The patient being under the influence of an anæsthetic, the iron, heated to whiteness, is gently pressed upon the part until a suitable eschar has been formed; one about the size of a twenty-five cent piece, if the patient be very young, or twice that size, if he be twelve or fifteen years old. The slough, which should not extend beyond the subcutaneous cellular tissue, will generally drop off within the week; and, during this period, as well as afterwards, the parts should be kept constantly covered with a linseed poultice, renewed several times in the twenty-four hours. The discharge, if flagging, may be promoted by savine ointment, by simple cerate containing a few drops of nitric acid to the ounce, or, what I prefer, the occasional application, for a few hours, of a small blister. In this manner I have known an abundant pyogenic discharge maintained for the greater part of a year. The cautery may, if necessary, be reapplied at any time during the progress of the treatment.

I give the hot iron a decided preference, for making an issue in this disease, to all other modes of cauterization, as affording not only a more copious and persistent flow of pus, but, what is of no little importance, making a much stronger, as well as a more permanent, impression upon the part and system. It is impossible always to determine how long the suppurative action should be kept up; but it will be found to be a good rule to let it continue until there is reason to believe that the morbid process, for the relief of which it was instituted, has completely subsided. I have never, in any instance, experienced any bad effects from its protracted continuance; indeed, quite the contrary has been the case. If the discharge becomes offensive, as it often does in warm weather, or where proper attention to cleanliness is not observed, the chlorides will come in play, with a more frequent change of dressings. Occasionally the linseed poultice oppresses by its weight, or it causes painful and itchy eruptions around the sore; when this happens it must be temporarily suspended, or be replaced by some more suitable application.

Independently of the advantages here alluded to, as being afforded by the issue made with the hot iron, there is another of nearly equal, if not, indeed, greater value. I allude to the fact that it may be used most efficiently for the local application of morphia, not so much for inducing sleep, as with a view of allaying the excessive pain of the parts, and the spasmodic jerkings of the muscles of the limb. Employed in this wise it acts, according to my observation, much more promptly and energetically than when administered by the mouth. The quantity necessary to produce the desired effect will, of course, depend upon circumstances; but, in general, from half a grain to a grain will be required for a child three or four years old. Sometimes an injection of laudanum, or an opiate suppository, will allay the pain and quiet the system more effectually than anything else.

In regard to the patient's diet, in this stage of the disease, the same rules should govern us as in the preceding stage; but it should, if possible, be somewhat more restricted, especially if there be much pain, with occasional fever. The bowels should also be maintained in an active condition, the best general purgative being a few grains of blue mass, with a suitable quantity of jalap, or the compound calomel pill. Salines and nauseants will rarely be required. If the patient labors under symptoms of debility, as denoted by the pallor of his countenance, the coldness of the extremities, and the disorder of the digestive organs, tonics, especially quinine and iron, should be employed, along with a more generous diet and nutritious drinks.

In the third stage, characterized by serious structural lesion, and the formation of matter, the treatment will require to be essentially modified, to meet the local and constitutional contingencies. Prior to this period, it was, if not decidedly antiphlogistic, at all events rather of a depressing character, intended to subjugate inflammation and establish resolution; now that the morbid action has gained the ascendency, and exhausted both part and system, a widely different course is demanded.

Two most important indications are presented, in almost every case, at this stage of the complaint. The first is to limit the suppurative process; the second, to promote the speedy absorption of the effused fluid. To fulfill either is generally no easy task; for experience has proved that there are few cases in which our efforts, however skilfully conducted, are crowned with success. When the suppurative process declares itself by well-marked symptoms, as rigors, alternating with flushes of heat, and followed by copious sweats, something may be done to moderate its violence by the steady and energetic use of antiphlogistics; but, in general, it will be found that the patient is either too feeble to justify their employment, or that the disease has been so insidious in its approaches as to defy recognition before the mischief has been completed. It has been seen, however, that matter does not always attend this stage, and that when it does, it often exists only in small quantity. Now it is in the latter class of cases, more particularly, and in those where the suppurative process can be carefully watched from its very inception, that strenuous and persistent efforts should be made to prevent its accumulation, with its concomitant mischief. For this purpose the affected hip should be painted at least twice a day with some sorbefacient lotion, such as equal parts of tincture of iodine and alcohol, or a weak solution of iodine and iodide of potassium, care being taken that, while applying it efficiently, it is not put on so freely as to cause pain or irritation beyond a few minutes. The object should be merely to excite the absorbents, the capillaries being fully kept in abeyance; otherwise injury, not benefit, will be likely to result. Sometimes a large blister answers a good purpose; retained just sufficiently long to produce slight vesication, and repeated every four or five days for several successive weeks. Thorough or protracted vesication is to be avoided, as likely to prove detrimental, from its irritating effects, both upon the part and system. If the disease has not yet advanced too far, if the abscess is still small, and, above all, if the suppurative process be attended with great pain and constitutional disturbance, I know of no remedy so well calculated to fulfill the present indication as the actual cautery, applied in such a manner as to produce a broad but superficial issue. My experience teaches me that, even in this stage of the malady, when everything is apparently most unpromising, more benefit will, in general, accrue from this mode of counter-irritation than from any other with which we are acquainted. It will not only, in many cases, promote the absorption of the pus that is already effused, but materially curtail the suppurative process, and at the same time greatly ameliorate the local distress. If the accumulation be very large, the remedy must, of course, be dispensed with, and artificial evacuation encouraged.

Along with the local remedies just pointed out, should be used certain internal means, to aid and expedite their sorbefacient action. At the head of the list may be placed mercury, especially the iodide and bichloride, administered in small quantities several times a day, and properly guarded by opium, to prevent them from griping and purging; the iodide of potassium and Lugol's solution are also valuable remedies. Ptyalism is of course avoided, and the strength is sustained with tonics, a generous diet, and nutritious drinks.

If these means fail, or if the abscess be already very large when we are called to the case, the matter should be evacuated artificially. I am aware that, upon this subject, there is generally a great deal of repugnance evinced by practitioners, on the supposition that any interference of this kind will only tend to aggravate the local disease, and cause hectic irritation. To such a view, however, I cannot subscribe. On the contrary, I can see no just reason why the general rules of surgery should be departed from in this disease any more than in others. It is well known that matter, wherever and whenever existing in large quantity, invariably does harm; for, not only is it apt to burrow extensively among the surrounding parts, but it must necessarily, by the pressure which it exerts upon the inflamed surfaces with which it is in contact, increase the pain, and effectually prevent the restorative process. That this is the case in regard to extensive purulent collections generally is a fact so well established as to require no argument in its support, and if there be any exceptions respecting such accumulations in the hip-joint I am not aware of them. My conviction is, that practitioners, influenced by the dogmas of the schools rather than by their personal experience and the dictates of common sense, have seriously erred in their treatment of strumous abscess of the ileo-femoral articulation, and been thus directly instrumental in the production of much mischief. In their endeavor to avoid too early an outlet, they have too frequently allowed themselves to wander into the opposite extreme, and neglected to make any opening at all: in other words, in attempting to steer clear of Scylla they have unwittingly rushed into Charybdis. The proper plan, undoubtedly, is, in all cases where the absorption of the matter is no longer a probable event, to promote its escape by the knife. It is the one which I have invariably pursued in all joint diseases for upwards of twenty years, and in no instance have I seen any reason to regret it. In this manner vast suffering is avoided, as well as much structural lesion prevented, as is abundantly shown by the numerous and tortuous sinuses which so frequently form in the hip when the disease is left to pursue its own course. The artificial opening, however, is not to be made heedlessly, or without due attention to the permanent exclusion of the air, which, although in itself innocuous, often proves pernicious, from its tendency, when brought in contact with the pent-up fluids, to favor decomposition, and, consequently, the development of hectic irritation. These effects may generally be prevented by giving the opening a valvular form, as in evacuating chronic abscesses in other situations, by making a puncture rather than an incision, and by closing the orifice, immediately after the operation, with adhesive strips, supported by a light compress and bandage. If the quantity of matter be unusually large, a portion only should be drawn off at a time, the process being repeated in a few days until the whole is removed; but ordinarily the sac may be emptied advantageously at the first operation.

As soon as the fluid has been evacuated, whether partially or completely, a full opiate should be administered, in order to prevent undue reaction; and, if the joint becomes painful, warm anodyne fomentations should be used, until relief is afforded. Afterwards, with a view of moderating suppuration, the parts are painted regularly once a day with some sorbefacient lotion, a linseed poultice or the warm water dressing being used as the permanent application.

If sinuses form, whether as a result of spontaneous or artificial evacuation, the best plan is not to interfere with them, as the pain and loss of blood consequent upon the employment of the knife would more than counterbalance any good effects from the operation. If loose fragments of bone, or pieces of cartilage, present themselves, they should, of course, be promptly extracted.

The violence of the suppurative action having been subdued, the plastic deposits are best dealt with by sorbefacient plasters, applied so as to embrace the whole of the affected surface, and steadily worn for many weeks. Of these plasters, the most useful, for this purpose, is the ammoniac and mercurial, under the influence of which the induration and swelling often disappear with astonishing rapidity. If much pain be present, a suitable quantity of morphia, opium, or cicuta may be incorporated with its ingredients.

During the latter part of this stage tonics and stimulants are usually indicated, and often imperatively demanded, by the exhausted state of the system. The patient, emaciated and anæmiac, must be supported with quinine and iron, cod-liver oil, a generous diet, and nutritious drinks, as milk-punch, wine-whey, ale, or porter. If night-sweats be present, the most suitable remedy will be the aromatic sulphuric acid, either alone or in union with Huxham's tincture of bark. Diarrhœa and pain must be checked with opiates. The patient's apartment should be frequently ventilated, and the surface of the body should be daily washed with tepid salt-water, followed by dry frictions. Exercise in the open air is now generally of much service, and may often be taken by the little patient himself, seated in a go-car, and propelled gently round the yard, or over a smooth road. Carrying him about in the arms is highly objectionable, on account of its tendency to hurt the joint, by pressing the affected limb. In a word, nothing should be omitted that may tend in the slightest degree to invigorate the system and improve the health.

When the extremity of the thigh-bone is necrosed, or so completely carious as to forbid all hope of recovery by time and ordinary means, the soft parts being riddled with sinuses, and the discharge copious and exhausting, excision of the diseased parts will be demanded, and should, if life be not too far exhausted, be promptly executed, as most likely to rescue the patient from impending death. The object of the undertaking, of course, is to remove all the ulcerated structures, and hence the surgeon, after having exposed the joint, should not limit himself merely to the thigh-bone, but perform a similar operation, if necessary, upon the margin and bottom of the acetabulum. The disease being thus arrested, the part and system will be placed in a much better condition for gradual and permanent recovery, provided the shock of the operation is not so severe as to destroy life, either immediately or consecutively. With proper care after the excision, it may even be possible to preserve a certain degree of motion between the contiguous bones. In an interesting and instructive paper on "Excision of the Head of the Femur, and Removal of the Upper Rim of the Acetabulum, for Morbus Coxarius," by Dr. Lewis Sayre, of New York, an analysis of thirty cases, including one by himself, is given, of which twenty recovered, and ten died, four of them within one week after the operation. It would thus appear that excision of this joint is by no means devoid of danger, although the mortality is much less than after amputation of the hip-joint. The statistics of Dr. Sayre have recently been extended by Dr. R. A. Kinloch, of Charleston, South Carolina, with no material variation, however, as to the results. In the case operated upon by himself, death occurred in less than thirty hours from exhaustion.

The operation may be conveniently performed by making a curved incision, from five to six inches in length, perpendicularly over the joint, in a line with the great trochanter, separating the parts, and cutting off the diseased structures with the saw or pliers. If more room be required, the incision may be crucial, or in the form of a T or V. The flaps are afterwards approximated with stitches and plasters, and the immobility of the limb is secured by appropriate splints, pads, and bandages, as in fracture of the thigh-bone.

When the patient has been exhausted by protracted suffering, and life is fast ebbing away, it has been proposed, as a dernier resort, to amputate at the hip-joint; and the records of surgery contain several examples in which the operation has been followed by the most gratifying results. Notwithstanding this, however, I should hesitate before undertaking so grave a procedure, the more especially as the same end may generally be more readily attained by the more simple and less dangerous operation of excision.

